# The complete chloroplast genome of *Castanea sativa*, an endemic to Europe

**DOI:** 10.1080/23802359.2021.1907246

**Published:** 2021-04-01

**Authors:** Haifeng Xu, Qi Zhang, Guifang Wang, Kun Xiang, Qingzhong Liu, Xin Chen

**Affiliations:** a Shandong Institute of Pomology, Shandong Academy of Agricultural Sciences, Taian, PR China; b Ji’nan Forest and Fruit Technology, Promotion and Industrial Service Centre, Ji’nan, PR China

**Keywords:** *Castanea sativa*, chloroplast genome, phylogenetic tree, Fagaceae

## Abstract

Sweet chestnut (*Castanea sativa*) is an endemic species of genus *Castanea* in Europe, which is widespread in the southern part of continental Europe. The complete genome sequence of chloroplast was determined through Illumina NovaSeq platform. Totally the genome of chloroplast was 160,938 bp in length, GC rich (36.8%), comprising a pair of 25,726 bp inverted repeat sequences, separated by a 90,519 bp large and 18,967 bp small single-copy regions. There were 129 genes, including 37 transfer RNA genes, 8 ribosomal RNA genes, and 84 protein-coding genes. The phylogenetic tree analysis showed that *C. sativa* exhibited the closest relationship with *Castanea henryi*.

Sweet chestnut (*Castanea sativa*) is a medium-large monoecious tree that is able to reach 30–35 m and has long-living (up to 1000 years), it is an endemic species of genus Castanea in Europe and is widely distributed in all Mediterranean countries (Fineschi et al. 2000; Conedera et al. 2016). Villani et al. (1991) showed that the genetic diversity in Turkey deme was greater than that in Italy and France, indicating that Turkey was the secondary origin and genetic diversity center of *C. sativa*. Some scholars thought that genus Castanea originated in Asian continent (Huang et al. 1994), and resulted in the *C. sativa* by westward migration (Zohary and Hopf 1983). Recent research found that Last Glacial Maximum (LGM) refugia of *C. sativa* were in the north of the Italian, Balkan Peninsulas, Iberian, and Anatolia, and the species spread naturally during the early-middle Holocene (Krebs et al. 2019). Roces-Díaz et al. (2017) showed a postglacial expansion of *C. sativa* in mid-Holocene at more favorable climatic conditions. At present, the complete chloroplast genomes of *Castanea seguinii*, *Castanea henryi*, and *Castaneamollissima* were all reported, however, the complete chloroplast genome of *C. sativa* is unknown. In this study, we annotated the chloroplast genome of *C. sativa* into GenBank public database with the accession MW044606.

The leaves of *C. sativa* were sampled from Taian chestnut garden of national fruit tree germplasm, Shandong province, China. The samples were kept in −80 °C refrigerator at Shandong Institute of Pomology, Taian, China. Genomic DNA of *C. sativa* leaves was extracted according to CTAB method (Doyle and Doyle 1987). The genomic DNA was performed a series of processing according to the standard protocol provided by Illumina company, including genomic DNA purification, library construction, library-quality detection, and paired-end sequencing on Illumina NovaSeq platform. The clean data were obtained from raw sequence data using trim_galore version 0.4.4 software (Babraham Institute, United Kingdom of Great Britain and Northern Ireland) and was aligned to genome of *C. mollissima* chloroplast (GenBank accession HQ336406) using bowtie2 version 2.2.4 software (Johns Hopkins University, America). The chloroplast genome was assembled by SPAdes version 3.10.1 software (St Petersburg State University, Russia), and all of the genes were annotated using GeSeq through comparison with the genome of *C. mollissima* chloroplast. The MISA version 1.0 software (MIcroSAtellite identification tool, http://pgrc.ipk-gatersleben.de/misa/misa.html) was used for analysis of simple sequence repeats (SSRs).

The length of chloroplast genome in *C. sativa* was 160,938 bp. It was a quadripartite structure including a 90,519 bp large single-copy and an 18,967 bp small single-copy, which were separated by a pair of 25,726 bp inverted regions. The total GC content of chloroplast genome in *C. sativa* was 36.8%. There were 129 genes, including 37 transfer RNA genes, 8 ribosomal RNA genes, and 84 protein-coding genes. In addition, the numbers of SSR were respectively 187 in single base repeat, 17 in double base repeats, 79 in triple base repeats, 11 in quad base repeats, 4 in five base repeats, and 1 in six base repeats.

In order to study the molecular phylogenetic relationship, the 35 chloroplast genomes of different species were selected to construct a neighbor-joining (NJ) tree using MEGA software version 6.0 (Auckland, New Zealand) and bootstrap analysis of 1000 replicates (Figure 1). The main species displayed on the phylogenetic tree were *Fagaceae*, *Betulaceae*, and *Juglandaceae*. In *Fagaceae*, there was a closer relationship between genus *Castanea* and genus *Castanopsis*. Further analysis indicated that *C. sativa* exhibited the closest relationship with *C. henryi*.

**Figure 1.**
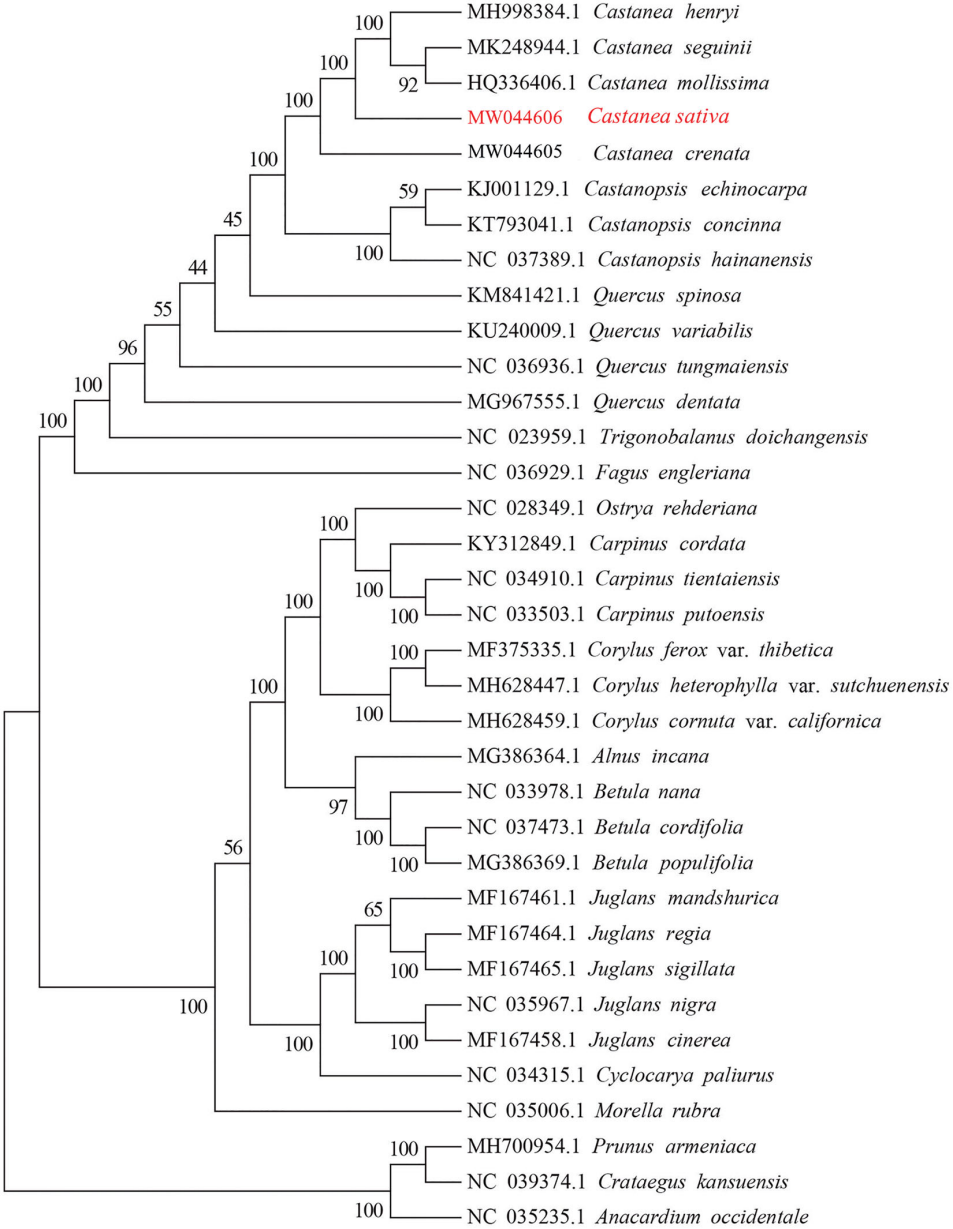
Phylogenetic tree based on 35 complete sequences of chloroplast genome in different species. The accession number in red font was the newly sequenced *Castanea sativa* in this study.

## Data Availability

The genome sequence data that support the findings of this study are openly available in GenBank of NCBI at (https://www.ncbi.nlm.nih.gov/) under the accession no. MW044606. The associated SRA number is SRR13649373.
